# Interferences between time and space in advanced age

**DOI:** 10.3758/s13421-025-01775-0

**Published:** 2025-08-12

**Authors:** Cindy Jagorska, Isa Steinecker, Martin Riemer

**Affiliations:** 1 Biological Psychology and Neuroergonomics, Technical University Berlin, Fasanenstr. 1, 10623 Berlin, Germany; 2 Bernstein Center for Computational Neuroscience (BCCN), Berlin, Germany; 3 Center for Behavioral Brain Sciences (CBBS), Magdeburg, Germany

**Keywords:** Aging, Time perception, Space perception, Space–time interference, Virtual reality

## Abstract

Perceptual interference between time and space has been reported in neonates, infants, children, and young adults, but to date it is unknown how space–time interference develops in advanced age. The presented study aims to bridge this gap by testing these interference effects in older (60 + years) and younger (18–35 years) participants. We asked our participants to reproduce the temporal duration or the spatial size of realistic three-dimensional (3D) stimuli (virtual rooms of different size presented in immersive virtual reality (VR)) and of abstract two-dimensional (2D) stimuli (squares presented on a PC screen). The results show that space judgments of older versus younger adults are more affected by irrelevant temporal information (time-on-space effect), whereas the reverse space-on-time effect was not significantly different between age groups. Space–time interference did not differ between 3D and 2D task versions. Together, our findings provide first insights into the development of space–time interference in advanced age.

## Introduction

In our daily lives we often encounter situations in which temporal and spatial information need to be integrated to correctly interact with the environment (e.g., catching a ball or crossing a busy street). Several studies have demonstrated that judgments regarding one of these dimensions (i.e., space or time) can be influenced by the other dimension (Bonato et al., 2012; Riemer & Cai, 2024). For example, large objects are perceived to endure for a longer duration (Xuan et al., 2007), longer time intervals between equidistant stimuli lead to an over-estimation of this distance (Helson, 1930), and in spatial navigation, the perception of traveled distance is influenced by travel time (Jagorska & Riemer, 2024). One theory accounting for such cross-dimensional interference postulates a common neuronal system to process general magnitude, encompassing the spatial and the temporal dimension, but also extending to other dimensions such as numerosity or brightness (Cona et al., 2021; Walsh, 2003). Neuroimaging studies frequently support the notion of such a general magnitude system located in the right intraparietal sulcus (Bueti & Walsh, 2009; Cona et al., 2023; Mock et al., 2018; Riemer et al., 2024). While the idea of a common magnitude system implies a symmetric interference between different magnitudes, there are many studies reporting asymmetric interference effects between space and time, mostly with time being more affected by space than vice versa (e.g., Casasanto et al., 2010; Casasanto & Boroditsky, 2008; Reali et al., 2019; Riemer et al., 2024; but see Bogon et al., 2025; Martin et al., 2017). One explanation accounting for such an asymmetric interference pattern is metaphoric structuring, which proposes that space exhibits a stronger influence on time than vice versa because we use spatial metaphors to think about time (e.g., “the future lies ahead”; Casasanto & Boroditsky, 2008; Lakoff & Johnson, 1980). An alternative explanation was offered by Cai and Wang (2022), who proposed that magnitude interference occurs due to representational noise in the stimuli. They argue that it is not the spatial representation per se that exerts a greater influence on the temporal representation, but rather the less noisy representation that exerts a greater influence on the noisier representation (see Fig. 1 in Riemer & Cai, 2024). Compared to space, time is often the noisier representation, because space–time interference is mostly investigated using visual stimuli, which promote spatial processing (Loeffler et al., 2018), whereas temporal processing is rather promoted by auditory stimuli (Handel, 1988; Ortega et al., 2014). The greater representational noise in the temporal versus the spatial domain is reflected in a heightened variability of temporal versus spatial judgments (e.g., Cai et al., 2018; Robinson & Wiener, 2021).

**Fig. 1:**
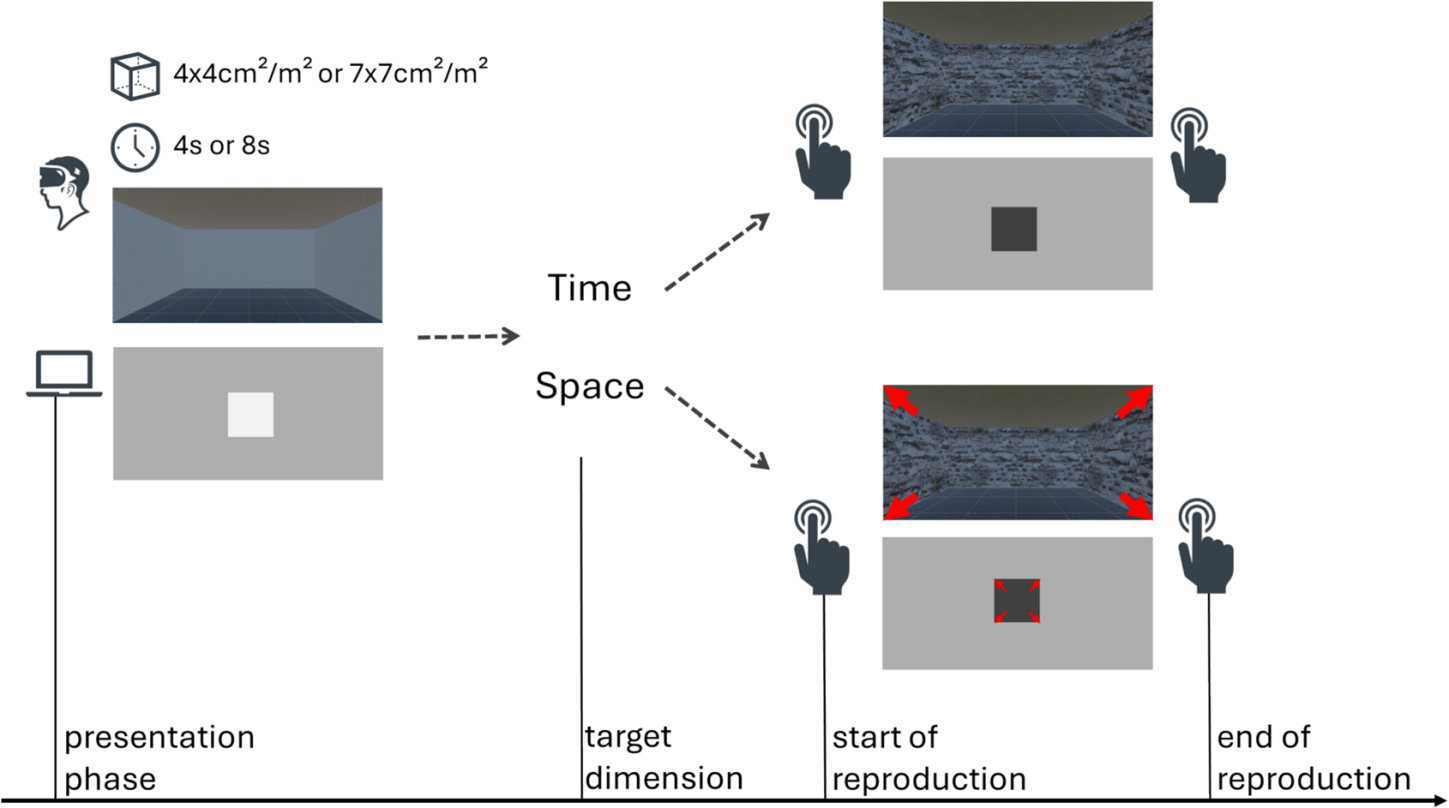
Trial structure for the time and space task. The upper images show examples for three-dimensional (3D) stimuli presented via a head-mounted display in immersive virtual reality (VR); the lower images show the two-dimensional (2D) stimuli. In the presentation phase, small or large stimuli were presented for a short or long duration. In time trials, a medium-sized stimulus had to be stopped after the same interval had elapsed. In space trials, a slowly expanding stimulus had to be stopped when it had reached the same size

While space–time interference is well studied in younger age groups ranging from young adults (Bratzke et al., 2024; Cai et al., 2018; Riemer et al., 2018; Xuan et al., 2007) to children (e.g., Bottini & Casasanto, 2013; Casasanto et al., 2010) and infants (e.g.,Lourenco & Longo, 2010; Srinivasan & Carey, 2010), and even to neonates less than 3 days after birth (e.g., de Hevia et al., 2014), there is a research gap when it comes to older adults. To date it is unknown how space–time interference develops in advanced age. This is unfortunate, because aging is accompanied by cognitive decline (Park et al., 2003), typically encompassing memory and attention processes (Yakhno et al., 2007), as well as spatial (Klencklen et al., 2012; Merhav et al., 2019) and temporal processing (Lustig & Meck, 2001; Maaß et al., 2019; Turgeon et al., 2016). Moreover, age-related cognitive decline is related to neuronal loss in the medial temporal lobe (Persson et al., 2012), a brain region that is critically involved in both spatial and temporal processing (Eichenbaum, 2017; Kraus et al., 2013; Montchal et al., 2019).

There have been various attempts to utilize age-related changes in temporal and spatial processing to predict cognitive decline (El Haj & Kapogiannis, 2016; Howett et al., 2019; Maaß et al., 2019; Mioni et al., 2021; Stangl et al., 2018). However, as primary deficits in time and space perception can be concealed by substitution strategies (e.g., chronometric counting), the assessment of space–time interference offers an elegant way of indirectly probing these deficits: If temporal (and spatial) processing abilities are compromised in advanced age and, as a result, the noise in temporal (or spatial) processing is high, this should lead to increased interference from the irrelevant spatial (or temporal) magnitude (Cai & Wang, 2022; Riemer & Cai, 2024). Consequently, cross-dimensional interference between space and time could be increased in advanced age, and might be one of the first behavioral signs of age-related cognitive decline.

When investigating interference between time and space, many studies utilize abstract two-dimensional (2D) stimuli (e.g., Cai et al., 2018; Martin et al., 2017; Schroeger et al., 2022; Xuan et al., 2007), which allow for a high level of control over the stimuli. However, abstract 2D stimuli may also lead to a reduction in ecological validity, as the most intuitive notion of space refers to the spatial extension surrounding our body (e.g., the size of a room). Also with respect to time perception, it was argued that the use of naturalistic stimuli increases the potential to assess changes in real-life timing behavior (Boltz, 2005; Maaß et al., 2022; Matthews & Meck, 2014; Riemer et al., 2021), and one way to achieve this goal is the presentation of stimuli in three-dimensional (3D) virtual environments instead of abstract 2D stimuli (Parsons, 2015). Some studies suggest differences in the representational noise of 3D versus 2D stimuli, although the direction of this difference depends on various factors. For example, in a mental rotation task, Neubauer et al. (2010) found increased performance for 3D versus 2D stimuli (suggesting a more stable representation of 3D objects), whereas Hughes (2001) reported reduced performance for judgments about the size of 3D versus 2D stimuli. Assuming that space–time interference depends on the representational noise of stimuli (Cai & Wang, 2022; Riemer & Cai, 2024), potential differences in the representational noise of 3D versus 2D stimuli should be reflected in different interference patterns.

To investigate how space–time interference evolves in advanced age, we asked younger and older adults for judgments on spatial size and temporal duration, while systematically varying the respective irrelevant dimension. We hypothesized bidirectional space–time interference, and a general increase of interference effects with advanced age. To assess the impact of a naturalistic 3D stimulus on space–time interference, we compared an immersive virtual reality (VR) set-up (subjects judged the size and duration of virtual rooms) with a more abstract version of the task involving 2D stimuli (subjects judged the size and duration of a white square presented on a PC desktop).

## Methods

### Participants

Forty-one older adults (self-reported gender: 29 female, 11 male, one non-binary; mean age: 67 years, ranging from 60 to 78 years) and 37 younger adults (self-reported gender: 17 female, 20 male; mean age: 27 years, ranging from 18 to 35 years) with normal or corrected-to-normal vision, participated in the study. Participants were recruited from the local community and the Technical University Berlin. They received either monetary compensation or course credits. All participants gave written informed consent to the experimental protocol. The study was approved by the local ethics committee of the Technical University Berlin (protocol number: MR_01_20200323).

Due to the lack of previous studies on which to base a simulation-based power analysis (Kumle et al., 2021), we aimed to recruit a minimum of 72 participants (36 per age group). For an alpha level of 0.05, this sample size ensured detection of an effect size of a η^2^_p_ = 0.10 with a power of 1 – β > 0.80 when using a classical ANOVA approach (calculated using MorePower 6.0; Campbell & Thompson, 2012). Two of the older participants did not complete the 3D task version (one because of reported motion sickness and one because of a technical issue).

### Experimental task and stimuli

A reproduction paradigm was used where participants were presented with a stimulus (presentation phase) which they then had to reproduce (reproduction phase). The to-be-reproduced stimulus was either of spatial magnitude (size) or temporal magnitude (duration). The task was presented on a PC display (2D task version) or in VR via a head-mounted display (3D task version). We employed the HTC Vive Pro kit (High Tech Computer Co),^1^ a VR head-mounted display with individually adjustable straps and adjustable interpupillary distance. The headset is compatible with glasses, an opportunity that was taken by some participants (from both the younger and the older groups). The order of task versions was counterbalanced across participants. Figure 1 shows a schematic depiction of the trial structure.

In the 3D task version, stimuli consisted of virtual rooms with a floor space of either 4 × 4 m^2^ or 7 × 7 m^2^ (room height was fixed to 3 m), with the participant sitting in the center of the room. The room was presented for either 4 or 8 s.^2^ After the presentation of the room (as a standard for this trial), participants were instructed to reproduce either its size or the duration for which it was presented. During the presentation phase, participants did not know which dimension (size or duration) they would have to reproduce. After a visual cue announcing this target dimension (“Space” or “Time”), participants pressed the space bar of a keyboard and started the reproduction phase. For duration reproductions, participants were presented with a medium-sized room (5.5 × 5.5 m^2^) and had to press the space bar again once the room had been visible for the same duration as the previously shown room. For size reproductions, participants were presented with a room of 1 × 1 m^2^ that continuously expanded around them. They were asked to press the space bar once they felt that the room had reached the same size as the previously shown room. To increase the discriminability between the presentation and reproduction phase, the wall textures of the rooms differed. While in the presentation phase the walls were always white, the wall textures in the reproduction phase differed (five different textures were used).

The trial structure in the 2D task version was analogous to the 3D task version (cf. Figure 1). Participants were presented with a white square the size of 4 × 4 cm^2^ or 7 × 7 cm^2^ that was shown for either 4 or 8 s. Both the 2D and the 3D task version contained 80 trials, presented in a randomized order, with participants being asked to take a break every 20 trials. Four practice trials were presented before each task version. No feedback was provided, in either in the practice trials or the main experiment. The 3D task version was presented on the HTC Vive Pro, the 2D task version was presented on a PC screen (59.5 × 33.5 cm^2^), with participants being seated approximately 65 cm from the screen.

### Assessment of memory capacity

We employed the *Verbaler Lern- und Merkfähigkeitstest* (Helmstaedter & Durwen, 1990), a German adaptation of the Auditory-Verbal Learning Test (AVLT; Ivnik et al., 1990). The procedure involves reading a list of 15 words to the participant five times. After each repetition, the participant is tasked with recalling these words from memory. Participants are asked to recall the words twice more, once after being presented with an interference word list (including 15 other words), and once after a delay of 30 min after the last recall. The exact procedure is described in Helmstaedter and Durwen (1990). As performance measures we report the total sum of correctly recalled words after the fifth repetition (Dg5), as well as the difference between the number of recalled words after the fifth repetition and after the 30-min delay repetition (Dg5-Dg7).

### Statistical analysis

For the analysis of space–time interference, we calculated the ratios between reproduced and standard values, so that values above 1 indicate over-reproduction and values below 1 indicate under-reproduction of duration or size, respectively. Outliers were defined within participants and conditions, using the median absolute deviation (MAD) procedure with a threshold of 3, as implemented in R package *Routliers* (Leys et al., 2013). In total, 3% of data were excluded based on this criterion. Individual space–time interference was quantified by taking the difference between the mean reproduced value of the task-relevant dimension (e.g., duration) when the task-irrelevant dimension (e.g., size) was small versus large. For example, a positive space-on-time effect would be indicated by a duration of a large stimulus being reproduced as longer than that of a small stimulus. We analyzed the data in a linear mixed-effects model (R package lme4 1.1–27.1; Bates et al., 2015) and fitted a 2 × 2 × 2 factorial design, including *age group* as a between-subjects factor (young vs. old), and *task type* (space vs. time) and *task version* (2D vs. 3D) as within-subjects factors. The isSingular() function in the lme4 package (Bates et al., 2015) indicated that mixed-effects models with random intercepts and slopes had a singular fit, suggesting overfitting or non-identifiable random effects. To improve model convergence and interpretability, we followed the suggestion of Bates et al. (2018) and adopted a more parsimonious model structure that included only random intercepts.

Additionally, the coefficient of variation (CV) was calculated for the ratios between reproduced and standard values. The relationship between response variability (as an indicator of representational noise; Cai & Wang, 2022) and space–time interference was assessed with two-tailed t-tests.

## Results

**Fig. 2:**
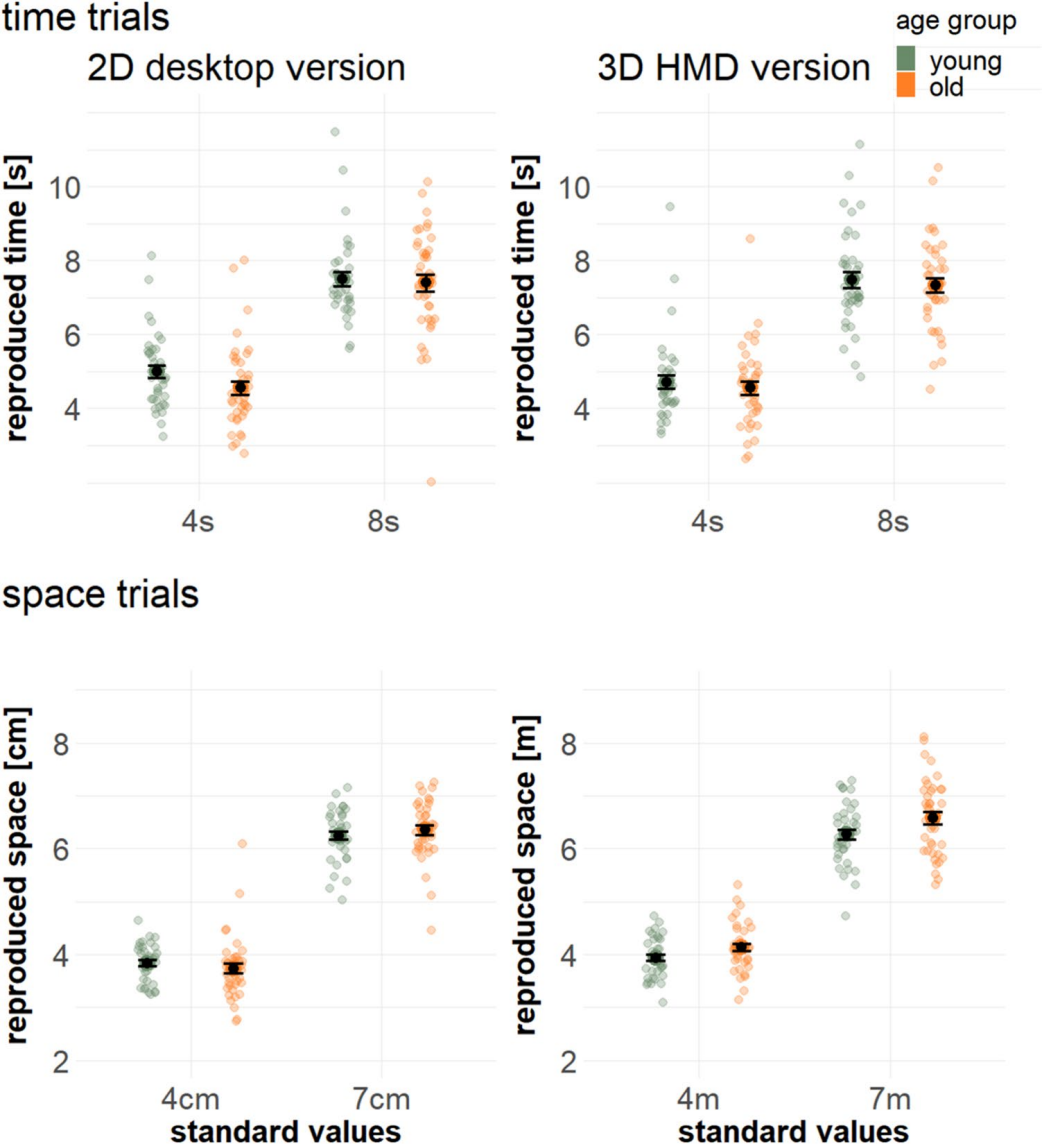
Reproduced values for time and space trials, as a function of task version, level of target dimension, and age group

**Fig. 3:**
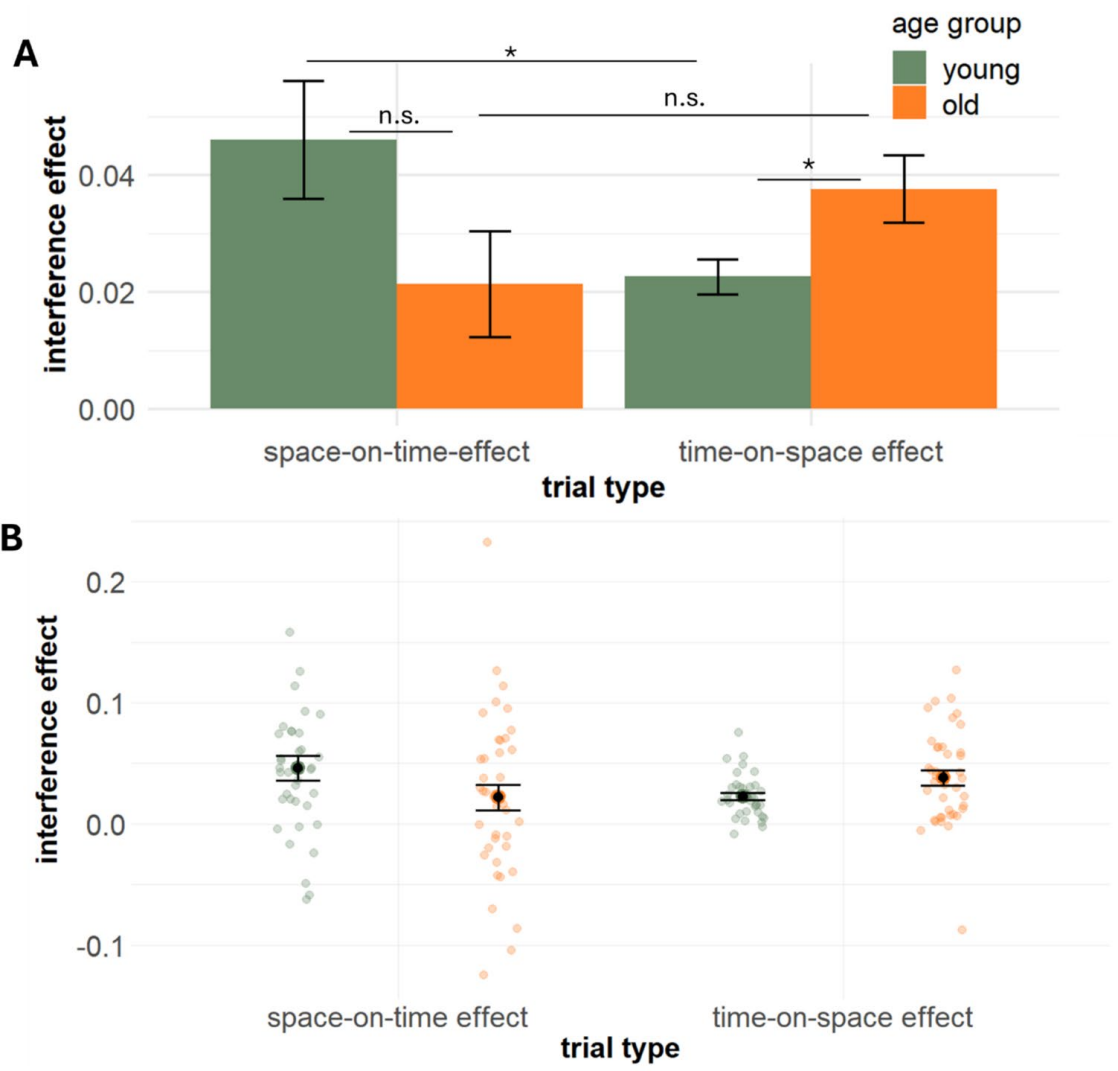
**(A)** Aggregated interference of space on time (**left**) and of time on space (**right**) as a function of age group (aggregated over *task version*). Error bars represent the mean ± standard error. **(B)** Individual interference of space on time (**left**) and of time on space (**right**) as a function of age group (aggregated over *task version*)

Figure 2 provides an overview of reproduced values in space and time trials. Regarding the analysis of interference effects, a significant main effect of the intercept (*β* = 0.03, SE = 0.003, t_73.0_ = 8.55, *p* < 0.001) indicates the general presence of interference effects. We found no significant main effect of *trial type* (*β* = −0.004, SE = 0.008, t_519.0_ = −0.493, *p* = 0.62), indicating no difference between the occurrence of time-on-space effects versus space-on-time effects. *Task version* (*β* = −0.008, SE = 0.008, t_519.0_ = −1.069, *p* = 0.29) and *age group* (*β* = −0.005, SE = 0.008, t_73.0_ = −0.660, *p* = 0.51) also did not show significant main effects. There was no significant interaction between *trial type* and *task version* (*β* = −0.008, SE = 0.015, t_519.0_ = −0.56, *p* = 0.57), and between *task version* and *age group* (*β* = −0.006, SE = 0.015, t_519.0_ = −0.46, *p* = 0.533). However, a significant interaction between *trial type* and *age group* (*β* = 0.04, SE = 0.015, t_519.0_ = 2.51, *p* = 0.009) indicates that younger adults exhibit a more pronounced time-on-space effect (as compared to the space-on-time effect), while older adults did not show this difference. This interaction is depicted in Fig. 3. T-tests confirmed that time-on-space effects were higher than space-on-time effects for younger adults (t_36_ = 2.2, *p* = 0.04), while the effects did not differ for older adults (t_40_ = −1.04, *p* = 0.31). Additionally, we observed a larger time-on-space effect for older as compared to younger adults (t_56.5_ = −2.2, *p* = 0.04), while the space-on-time effect did not differ between age groups (t_72.3_ = 1.5, *p* = 0.14).

**Fig. 4:**
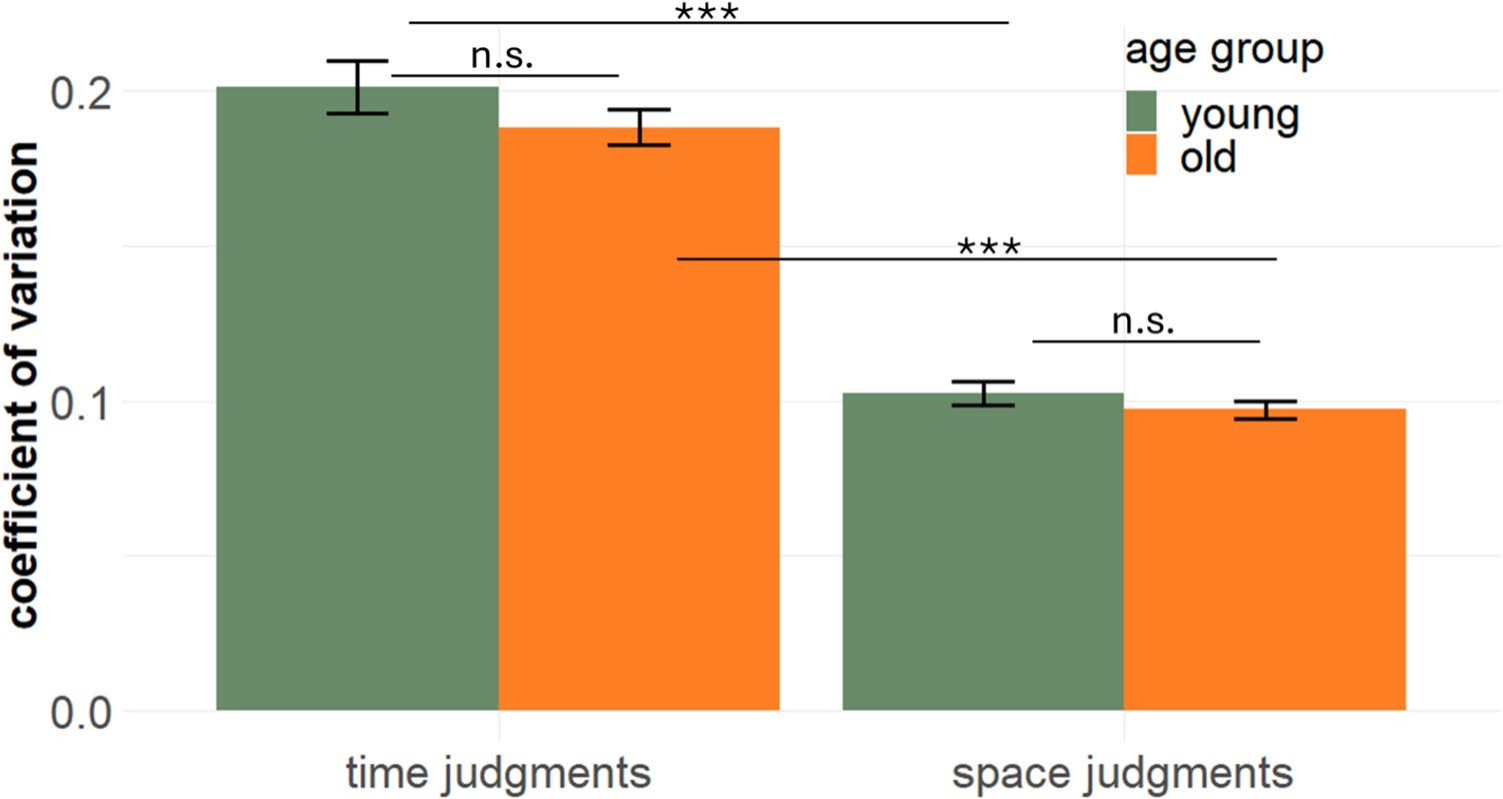
Response variability for time (**left**) and space judgments (**right**) as a function of age group (aggregated over *task version*). Error bars represent the mean ± standard error

Regarding the CV (Fig. 4), we found a higher variation in time as compared to space trials for younger (t_36_ = −14.1, *p* < 0.001) and older adults (t_39_ = −10.3, *p* < 0.001), but no difference between age groups within the trial types (time trials: t_67_ = −1.0, *p* = 0.34; space trials: t_71_ = −0.9, *p* = 0.37).

With respect to memory capacity, older adults performed worse than younger adults, both regarding the total sum of correctly recalled words after the fifth repetition (Dg5: *t*_73.95_ = 2.82, *p* = 0.006) and with regard to the difference between the number of recalled words after the fifth repetition and after the delayed recall (Dg5-Dg7: t_73.92_ = −3.02, p = 0.003). Note that older adults performed within the normal range for their age group with regard to both measures (Dg5: mean: 55.44, SD = 8.21; Dg5-Dg7: mean: 1.7, SD = 1.9; Volz-Sidiropoulou et al., 2010).

## Discussion

Space–time interference has been consistently observed in humans of various ages, ranging from neonates (e.g., de Hevia et al., 2014), early infancy (e.g., Lourenco & Longo, 2010; Srinivasan & Carey, 2010), and childhood (e.g., Bottini & Casasanto, 2013; Casasanto et al., 2010) to adults (e.g., Cai et al., 2018; Riemer et al., 2018), but to date it is unknown how these effects develop in advanced age. In the present study, we tested interference between spatial size and temporal duration in younger (18–35 years) and older (60 + years) adults, using naturalistic 3D stimuli presented in immersive VR as well as abstract 2D stimuli presented on a PC screen. Both age groups demonstrated significant bidirectional interference between space and time. Moreover, the often-reported asymmetry in space–time interference (with time judgments being more affected by space than vice versa; Casasanto & Boroditsky, 2008) was only confirmed within the younger group. While for older adults no significant difference was found with respect to the direction of interference, they showed a stronger influence of time on space as compared to younger adults (cf. Figure 2). With respect to the space on time effect, no age-related differences were found. These results complement previous research by providing first evidence that space–time interference changes in advanced age, depending on the direction of influence.

It has been proposed that the direction of space–time interference (and cross-dimensional interference in general) depends on the relative representational noise in the interfering dimensions (Cai & Connell, 2015; Cai & Wang, 2022; Riemer & Cai, 2024). Less noisy representations should exert a larger influence on more noisy representations. For example, Cai and Wang (2022) asked their participants to judge spatial lengths that were either presented statically (distance between two simultaneously presented bars) or dynamically (distance between two sequentially presented bars). They found that the influence of time on length judgments was stronger when length was dynamically presented, that is, when representational noise was arguably larger. With respect to the present study, this idea of an influence of representational noise can explain the asymmetry of space–time interference in younger adults: The variability of time judgments was larger than that for space judgments (indicating higher representational noise), and therefore time was more influenced by space than vice versa. This finding is also in line with studies suggesting that higher variance in time judgments is the cause of asymmetric space–time interference (Cai et al., 2018; Riemer & Cai, 2024). However, the idea conflicts with the observation of a larger time-on-space effect for older versus younger adults, because no age-related differences in response variability were found, for either time or space judgments. Hence, the age-related difference in the time-on-space effect cannot be explained on the basis of representational noise alone. The finding of a more pronounced effect of time-on-space in older versus younger adults is also noteworthy given that previous studies on space–time interference typically report time as being more affected by space than vice versa (Casasanto & Boroditsky, 2008; Casasanto et al., 2010; Reali et al., 2019; Riemer et al., 2024).

A potential explanation is suggested by Martin et al. (2017), who stressed the importance of the relative informativeness of the spatial and temporal dimensions for each other (see also Lambrechts et al., 2013). In their experimental paradigm, the spatial magnitude progressively increased over time, thereby introducing a temporal component into the perception of space. This approach resulted in a more pronounced time-on-space effect (of reversed direction, that is, longer durations led to an underestimation of space) relative to the space-on-time effect. This finding underscores the role of accumulation over time for the processing of spatial magnitudes. During the reproduction phase in our study, the spatial task required participants to stop the expansion of either a 2D square or a 3D virtual room, thereby also introducing a temporal component. Conversely, the time reproduction task involved a 2D square or 3D virtual room of constant size and therefore did not include progressive changes of the irrelevant spatial information. Hence, similar to the studies by Lambrechts et al. (2013) and Martin et al. (2017), the present design might have accentuated the time-on-space effect, because temporal information was more informative for the space task than vice versa. The fact that this accentuated time-on-space effect is more prominent in older adults might reflect an increased difficulty to ignore the (irrelevant) temporal component in the spatial reproduction task. Indeed, there are several studies demonstrating age-related problems in suppressing irrelevant information (Girelli et al., 2001; Juncos-Rabadán et al., 2008), consistent with the inhibitory deficit hypothesis (Hasher & Zacks, 1988; Lustig et al., 2007). It remains unclear whether an age-related increase of time-on-space effects would also show in other types of reproduction tasks.

One might argue that the observation of age-related increases in space–time interference selectively for the time-on-space effect, but not the time-on-space effect, can be partially explained by age-related differences in the tendency to use chronometric counting strategies. Older adults can be reluctant to admit cognitive deficits and might adopt compensatory strategies to enhance their timing performance, inhibiting the effect of space on time. For example, Perbal et al. (2002) found that older participants performed equally to younger participants in a time-reproduction task when chronometric counting was permitted, but their performance significantly declined relative to the younger group when counting was disrupted by a concurrent distractor task. In this vein, a reduced space-on-time effect in older adults might result from the use of counting strategies. However, this explanation is not supported by our data, because no age-related differences in the variability of reproduced durations were observed. Hence, we consider it unlikely that our results can be explained on the basis of increased chronometric counting in older adults. It should be noted, however, that older as well as younger adults showed higher response variability in the timing task compared to the spatial task, which has also led to a heightened variability of the space-on-time effect compared to the time-on-space effect (see Fig. 3B). This might have obscured potential age-related differences in the space-on-time effect (cf. Figure 3).

Changes in temporal as well as spatial perception have been associated with pathological aging (Gazova et al., 2012; Howett et al., 2019; Mioni et al., 2024; Riemer, 2024; Turgeon et al., 2016). However, as primary deficits in time and space perception could be concealed by substitution strategies (e.g., chronometric counting; Rattat & Droit-Volet, 2012; Riemer et al., 2022a, 2022b), space–time interference provides an indirect way for detecting these deficits. If temporal and spatial processing is defective and the noise of the relevant dimension is high, this might lead to an increased influence of the irrelevant dimension and ultimately to more pronounced cross-dimensional interference.

Due to the prevalent use of abstract 2D stimuli in investigating space–time interference (e.g., growing lines, dots moving across a PC screen), another objective of the present study was to examine how these findings generalize to more naturalistic 3D stimuli, which might be more suited to assessing changes in real-life behavior and perception (Bogon et al., 2024a, 2024b; Boltz, 2005; Maaß et al., 2022; Matthews & Meck, 2014; Riemer et al., 2021; Thanopoulos et al., 2018). Here we found no differences in space–time interference between a 3D and a 2D task version, suggesting that naturalistic 3D stimuli do not affect the interference between temporal duration and spatial size. This validates the use of naturalistic 3D stimuli presented in immersive VR for the investigation of space–time interference, while it also confirms that reports of space–time interference for 2D stimuli are not solely based on the processing of relatively simplistic stimulus material. As the use of 3D stimuli primarily relates to the spatial domain, it is notable that the time-on-space effect was comparable in the 2D and 3D task versions. This may be due to the inherent temporal component of the spatial reproduction task. However, in other paradigms where size is reproduced non-continuously, the time-on-space effect may differ between the 2D and 3D task versions.

In accordance with the fact that the use of 3D stimuli primarily concerns the spatial domain, stimulus type only affected the reproduction of spatial size. The size of 2D stimuli was over-reproduced relative to 3D stimuli. Concerning the reproduction of temporal duration, we did not find an influence of naturalistic 3D stimuli. This is in line with other work, suggesting that immersive VR set-ups do not alter the perception of time per se (Bogon et al., 2024a, 2024b; van der Ham et al., 2019).

## Conclusion

This study provides first insights into the development of space–time interference in advanced age. Specifically, the effect of irrelevant temporal information on spatial judgments is found to be increased in older adults (compared to younger adults). This extends the existing literature, which has primarily focused on space–time interference in an age range from infancy to middle age. Additionally, our findings demonstrate that space–time interference persists across both abstract 2D and more naturalistic 3D task versions.

## Authorship contribution

**CJ**: Formal analysis, investigation, data curation, writing—original draft, writing—review and editing, visualization. **IS**: Software, investigation, data curation, writing – original draft. **MR:** Conceptualization, methodology, resources, writing – review and editing, supervision, project administration, funding acquisition.

## Data Availability

The data and scripts for all experiments are available at: https://github.com/cindyjag/TimeSpaceAdvancedAge.

## Footnotes

1 VIVE Pro Specs & User Guide: https://developer.vive.com/resources/hardware-guides/vive-pro-specs-user-guide/

2 Note that participants were not informed of the binary nature of the standard stimuli. Post-experiment ratings indicated that 95% of participants in the 2D time trials and 93% of participants in the 3D time trials rated the number of presented standard durations as “3 or more”. Regarding space trials, 83% of participants in the 2D version and 84% of participants in the 3D version estimated “3 or more” levels of standard sizes.

## Code availability

The experimental code can be shared upon request.
